# The brain of the North American cheetah-like cat *Miracinonyx trumani*

**DOI:** 10.1016/j.isci.2022.105671

**Published:** 2022-11-25

**Authors:** Borja Figueirido, Alejandro Pérez-Ramos, Anthony Hotchner, David M. Lovelace, Francisco J. Pastor, Paul Palmqvist

**Affiliations:** 1 Departamento de Ecología y Geología, Facultad de Ciencias, Universidad de Málaga, 29071 Málaga, Spain; 2 Anatomy Department, Des Moines University, 3200 Grand Avenue, Des Moines, IA 50312, USA; 3 University of Wisconsin-Madison, Department of Geoscience, Madison, WI 53706, USA; 4 Departamento de Anatomía y Radiología, Museo de Anatomía, Universidad de Valladolid, 47005 Valladolid, Spain

**Keywords:** Zoology, Evolutionary biology, Paleobiology

## Abstract

The cheetah *Acinonyx jubatus,* the fastest living land mammal, is an atypical member of the family Felidae. The extinct feline *Miracinonyx trumani,* known as the North American cheetah, is thought to have convergently evolved with *Acinonyx* to pursue fast and open-country prey across prairies and steppe environments of the North American Pleistocene. The brain of *Acinonyx* is unique among the living felids, but it is unknown whether the brain of the extinct *M. trumani* is convergent to that of *Acinonyx*. Here, we investigate the brain of *M. trumani* from a cranium endocast, using a comparative sample of other big cats. We demonstrate that the brain of *M. trumani* was different from that of the living *A. jubatus*. Indeed, its brain shows a unique combination of traits among living cats. This suggests that the case of extreme convergence between *Miracinonyx* and its living Old World vicar should be reconsidered.

## Highlights

• The brain of *Miracinonyx* exhibits a unique pattern of gyri and sulci
• The anterior cerebrum of *Miracinonyx* is not as reduced as the one of the cheetah
• The brain shape of *Miracinonyx* is not as dorsiflexed as the one of the cheetah
• The brains of the cheetah and *Miracinonyx* are not as convergent as expected

## Graphical abstract

**Figure:**
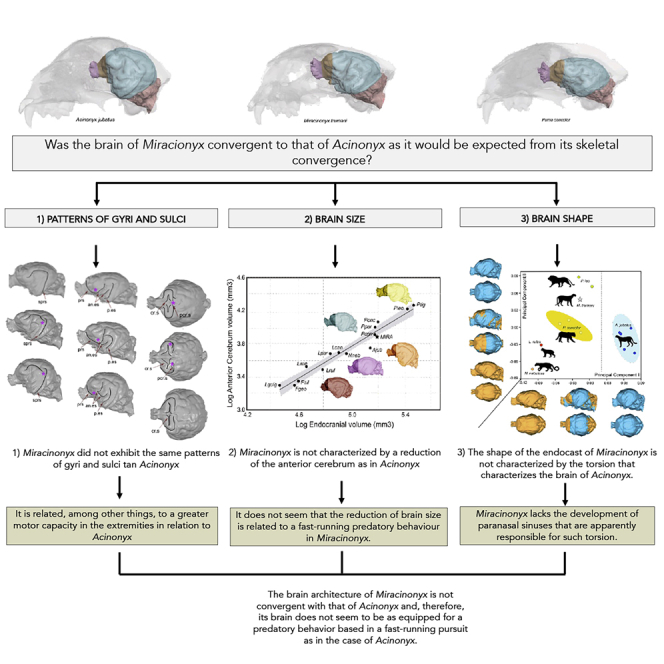

## Introduction

The living cheetah (*Acinonyx jubatus*) is widely acknowledged as the fastest living land mammal capable of speeds up to 25.9 ms^−1^ and reaching top accelerations in only 3 s.^1^ Most felids are stalking predators in closed habitat that rely on stealth to approach their prey, followed by a brief, high-speed pursuit.^2^ Although they can accelerate rapidly, they tire quickly and their attacks are often aborted when prey detects them during ambush or early in their approach.^3^ The only exception to this hunting behavior among the living felids is the cheetah, a diurnal, coursing predator with a highly specialized anatomy and physiology that relies on rapid acceleration and sprinting^4^^,^^5^^,^^6^ over chases of some hundred meters (average run distance: 173 ± 116 m^1^). In contrast to stalkers, the cheetah approaches its prey in open habitat with little or no stealth, surveying moving animals for weaknesses, chasing its prey at high speeds and killing it by strangulation.^7^^,^^8^^,^^9^

Several osteological and physiological adaptations of *A. jubatus* are thought to be related to an extremely fast chase-based predatory behavior.^10^^,^^11^^,^^12^ For example, this species has a highly specialized postcranial skeleton with a number of morphological features that are unique among felids, including the presence of elongated distal limb bones, interphalangeal elastic ligaments, ridged digital pads and lack of fully-retractable claws.^13^^,^^14^ Indeed, the generic name *Acinonyx* (from the Greek A-, ‘not’ + kinéō, ‘I move’ +-ónux, ‘claw’), means ‘immobile claws’ referring to the lack of claw hyper-retraction seen in other felids.^15^ The lack of fully retractable claws in *A. jubatus* is thought to be the result of relaxed selective pressures for the protection of the claws from blunting because they are protracted to gain traction during the high-speed chase.^14^^,^^16^ Moreover, the reduced ability of *A. jubatus* to manipulate prey is because of its low degree of forepaw dexterity, which ranks among the lowest of felids,^17^^,^^18^ mainly because of its reduced ability to supinate their forelimbs.^2^^,^^19^^,^^20^^,^^21^ As a result, *A. jubatus* does not manipulate prey with their forepaws.^18^ Instead, it causes the prey to lose its balance using its sharp dewclaws on the animal’s skin and shifting its own weight backwards.^14^^,^^22^^,^^23^

These adaptations appear to have evolved to capture small-to-medium sized prey such as Thomson gazelle (*Eudorcas* *thomsonii*), which can be subdued with minimal risk of injury and consumed rapidly before surrendering it after the arrival of kleptoparasites.^12^ Moreover, *A. jubatus* possess a reduced skull mass with lightened bone and elongated internal nostrils. This allows it to take a greater volume of air, which is necessary for aerobic exercise during prey chase.^24^^,^^25^ Not surprisingly, the cheetah shows a greater nasal aperture area than expected from its cranium and palatal dimensions, which evidences a greater breathing capacity compared to other felids,^26^ and shows also the densest packing of maxilloturbinates among the living cats.^27^ This is expected in a coursing predator that subdues prey after a prolonged chase, which requires increased breathing capacity for cooling the body during pursuit and compensating the oxygen deficit produced by the huge muscular efforts.^28^ Moreover, the cheetah’s cranium shows a greater interorbital breadth than a pantherine cat of similar size. A wide braincase is a condition typical of small felids^29^ and it appears that despite increasing its size to that of a pantherine felid, *A. jubatus* retained small-cat cranial proportions as it is paedomorphic.^28^

Compared to the pantherine felids, the cheetah shows some derived features, including (1) a domed and rostro-caudally compressed cranium; (2) a lateral enlargement of the frontals caudally to the zygomatic processes; (3) a widening of the nares and orbits, with the latter oriented frontward; (4) less developed sagittal and nuchal crests; (5) bowed zygomatic arches; (6) slender canines and narrow cheek teeth; and (7) a marked reduction of the protocone in the upper carnassial. In contrast, these features are less marked in the skull of the giant (∼100 kg) cheetah *Acinonyx pardinensis* from the Eurasian Pleistocene, which suggests that the highly derived skull shape of the modern cheetah probably evolved recently.^24^^,^^28^^,^^30^^,^^31^

The few postcranial remains available of *A. pardinensis* (e.g., a nearly complete foreleg from Dmanisi^32^) are suggestive of body proportions similar to the living cheetah (i.e., a slender skeleton with elongated limb segments). This led to the assumption of a direct similarity between the hunting strategy in *A. pardinensis* and *A. jubatus*, which would be based on a high-speed chase of small-to-medium sized prey.^32^ However, the musculoskeletal skull morphology of *A. pardinensis* suggests that it could catch larger ungulate prey through a killing strategy more similar to the extant pantherine cats than to the cheetah.^24^ Moreover, a recent study on the inner ear morphology^33^ in both *A. jubatus* and *A. pardinensis* suggests that the extinct form did not possess the distinctive attributes of the *A. jubatus* inner ear –i.e., greatest volumes of the vestibular system among cats, a dorsal extension of the anterior and posterior semicircular canals– that presumably correlate with a greater afferent sensitivity of the inner ear to head motions, facilitating postural and visual stability during the high-speed chase.^33^

On the other hand, brain overheating is thought to be a limiting factor for chase distance in the cheetah.^6^^,^^34^ The large frontal sinuses of *A. jubatus* are highly vascularized, which is thought to provide a cooling mechanism to prevent overheating of the brain during periods of high exertion.^25^^,^^35^ At the end of a sprint, body temperature reaches ∼38.5–41°C.^6^^,^^10^^,^^36^ After a chase, the cheetah is so exhausted that as many as 30 min may be elapsed catching its breath before it can eat.^9^

On the other hand, *A. jubatus* also possesses elongated limbs with a reduced muscle mass, which allow it for exerting both faster bursts of speed and longer stride lengths during running.^37^^,^^38^^,^^39^ In addition, a very flexible lumbar spine allows greater extension of the posterior back in the cheetah, which facilitates the aerial and land pose during the gallop phase^37^ and also increases stride length by 5% and top speeds by 10%.^10^^,^^40^ The tail of the cheetah can rotate across the horizontal and vertical planes, which allows it to perform conical movements around the sagittal axis of the body to function as a rudder or counterweight. This allows the animal to change its running direction while maintaining balance, and also helps it to prevent skidding and improve aerodynamics.^41^

The fossil record attests that this ‘built-for-speed’ design has appeared at least twice within felids: in the Old-World *A. jubatus* and also in the ‘cheetah-like’ cat *Miracinonyx* spp. from the North American Pleistocene. As evidenced by both molecular^42^ and morphological^43^ data, the closest living relative of *Miracinonyx* is the cougar (*Puma concolor*). However, its skeletal anatomy is extremely ‘cheetah-like’,^44^^,^^45^ at least in the more derived *M. trumani* from the late Rancholabrean.^43^ The earliest species ascribed to the genus *Miracinonyx* is *M. inexpectatus*, which is morphologically different than the more derived *M. trumani*.^43^*M. inexpectatus* differs from *M. trumani* in a set of traits that evidence a lesser degree of ‘cheetah-like’ specialization compared to the more recent species, including an overall larger size, a longer third upper premolar relative to the fourth upper premolar, a larger protocone in the fourth upper premolar, larger upper canines relative to the upper fourth premolar, smaller nasal aperture area, a stouter distal ulna, a lower brachial index (i.e., ratio of radius length to humerus length), and a less elongated patella.^43^

It is hypothesized that an ancestral form of *Puma*-*Miracinonyx* probably originated in the Old World, migrated to North America about 6 million years ago (Myrs) and gave rise to both *Miracinonyx* and *Puma*,^24^^,^^43^, (but see ref.^46^) about 4.0 Myrs –a date of divergence that is likely based on the earliest *Puma* records in North America (*P. lacustris*) from the Glenns Ferry Formation of Idaho.^47^^,^^48^ However, this same site also has preserved a single specimen that has been referred to *M. inexpectatus*, which is likely the oldest record of that species. Therefore, the occurrence of both taxa at a site spanning 4.18–3.11 Ma suggests the early divergence the *Miracinonyx* and *Puma* lineages.^47^^,^^48^

Based on this evidence, the most parsimonious explanation is that the ‘cheetah-like’ morphology of *M. trumani* is a result of convergent evolution with *Acinonyx* for fast-pursuit of prey across the prairies and steppe terrains of North America during the Pleistocene.^42^^,^^43^ Moreover, it has been proposed that Pronghorn ‘antelope’ (*Antilocapra americana*), the second-fastest modern land mammal with no natural predators that come close to matching their speed, would be the preferred prey of *M. trumani*.^49^

Further insights on the predatory behavior of *M. trumani* have been derived from analyses of stable-isotopes abundance of fossil collagen from Natural Trap Cave specimens*.* The trophic enrichment between herbivores and carnivores in collagen isotopes is 1.6‰ for carbon and 3.9‰ for nitrogen, respectively.^50^ In the case of Natural Trap Cave, the differences in δ^13^C and δ^15^N values for the single specimens analyzed by McNulty et al.^51^ of *M. trumani* and *A. americana* are close to those expected for a carnivore and its prey. However, this also applies to two of the four specimens of bighorn sheep (*Ovis canadensis*) analyzed, which opens the possibility that *M. trumani* also preyed on sheep in rocky environments. A more in-depth study of these fossils has been recently performed by Higgins et al.^49^ The mean δ^13^C and δ^15^N values obtained for two specimens of pronghorn (−18.7 and 4.7‰, respectively) and five of *M. trumani* (−17.3 and 7.9‰, respectively) are close –but slightly lower than expected– to the isotopic enrichment from prey to predator. Higgins et al.^49^ confirmed with an isotopic mixing model that pronghorn was the primary prey of *M. trumani,* contributing to 40% of the diet of the North American cheetah, and that other herbivores such as horse, bison, and sheep were also preyed on.

**Figure 1:**
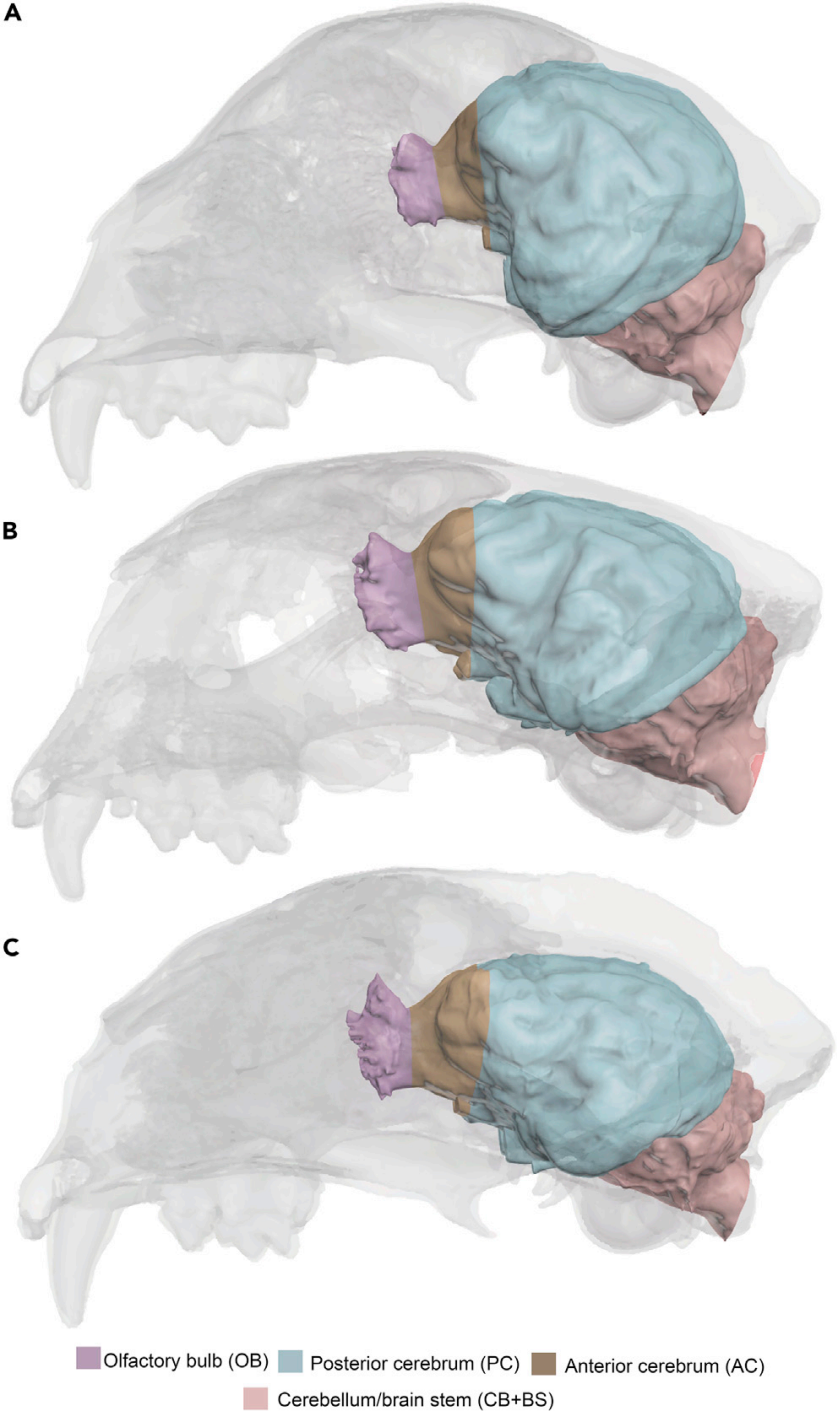
Brain endocast segmentation Some crania analyzed are taken as an example. (A) *Acinonyx jubatus*. (B) *Miracinonyx trumani*. (C) *Puma concolor*. Brain regionalization is shown in colors following previous studies.^55^

Although skeletal convergence between *M. trumani* and *A. jubatus* has been widely investigated,^43^^,^^45^^,^^52^^,^^53^^,^^54^ it is unknown whether the brain architecture of *M. trumani* is also convergent to that of *A. jubatus*. The brain of *A. jubatus* is unique among felids in gyral and sulcal patterns, as well as in regional size and shape, features that have been interpreted as related to its specialized predatory behavior.^55^^,^^56^^,^^57^ Here, we investigate gyral and sulcal patterns of the brain of *M. trumani,* and also the regional size and shape of functional brain areas from an endocast of a cranium preserved at Natural Trap cave (Northern Wyoming), and compare it quantitatively with a sample of living cats, including its Old-World vicar (*A. jubatus*) and its closest living relative (*P. concolor*) (Figure 1). Our main aim is to ascertain whether the brain of *M. trumani* also possesses the distinctive features present in *A. jubatus* that are thought to be related to its unique predatory behavior. Our initial hypothesis is that brain architecture of *M. trumani* is similar to that of *A. jubatus* given its close skeletal convergence toward a fast-running-chase predatory behavior.

## Results

### Patterns of gyri and sulci in *M. trumani*

**Figure 2:**
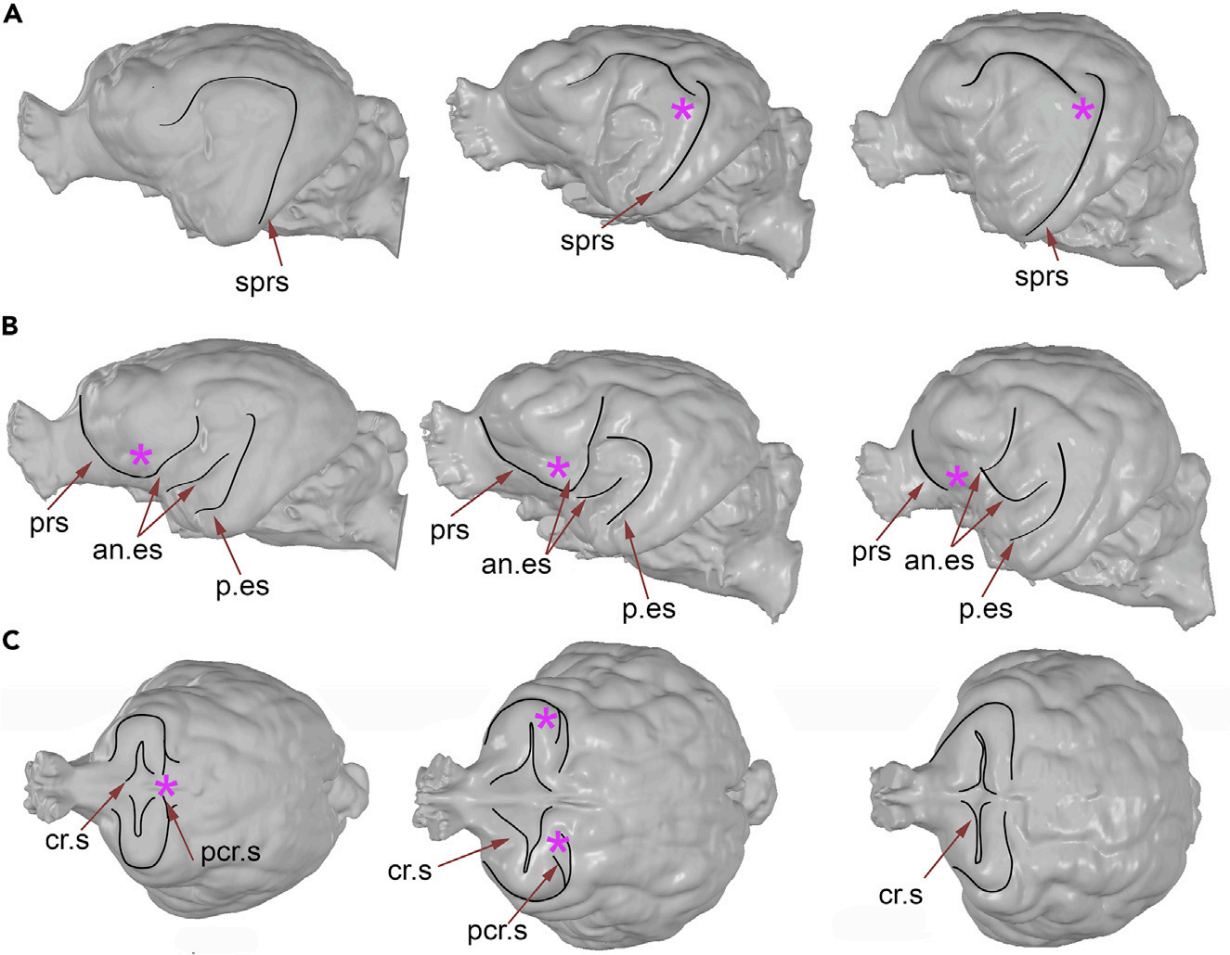
Patterns of gyri and sulci in *P. concolor* (left column), *M. trumani* (middle column) and *A. jubatus* (right column) (A) Lateral view of brain endocast showing the pattern of suprasylvian sulcus arch at the caudomedial corner. (B) Pattern of location of the orbital sulcus and ectosylvian sulcus. (C) presence or absence of postcruciate sulcus. Abbreviations: sprs, suprasylvian sulcus; prs, presylvian (orbital) sulcus; an.es, anterior ectosylvian; p.es, posterior ectosylvian; cr.s, cruciate sulcus; pcr.s, postcruciate sulcus. Asterisks denote areas of discussion through the text. See also Figure S1.

Following a previous study^57^, the gyral and sulcal patterns of the brain of modern felids is highly conservative, as they do not vary from the largest pantherine felids (*Panthera tigris* or *Panthera leo*), with cranial capacities of 250–300 cm^3^, to the smallest felid species (e.g., *Prionailurus rubiginosus*), with brains of 20–25 cm^3^. Moreover, there are not significant differences in regional brain proportions between the largest and smallest species.^57^ However, one notable exception to this pattern is the brain of *A. jubatus*. For example, its suprasylvian sulcus arch at the caudomedial corner –where the posterior and middle portions of the suprasylvian sulcus join– is disrupted. Our brain endocast data suggest that the suprasylvian sulcus is neither disrupted in *P. concolor* nor in *M. trumani,* although the continuation of this sulcus in the latter is more subtle (Figure 2A).

Unlike other felids, the orbital sulcus of *A. jubatus* does not continue with the ectosylvian sulcus, a disruption that is also observed in the *Lynx*.^57^ The endocast of *M. trumani* evidences a continuation of the orbital (presylvian) sulcus with the ectosylvian one, although by a very smooth sulcus area (Figure 2B).

The postcruciate sulcus of felids is plainly marked irrespective of brain size,^57^ but in *A. jubatus* it is usually absent or, at most, is reduced to a small dimple. Our brain endocast data indicates that the postcruciate sulcus is still present in *M. trumani* as a small dimple, as well as in *P. concolor* (Figure 2C).

**Figure 3:**
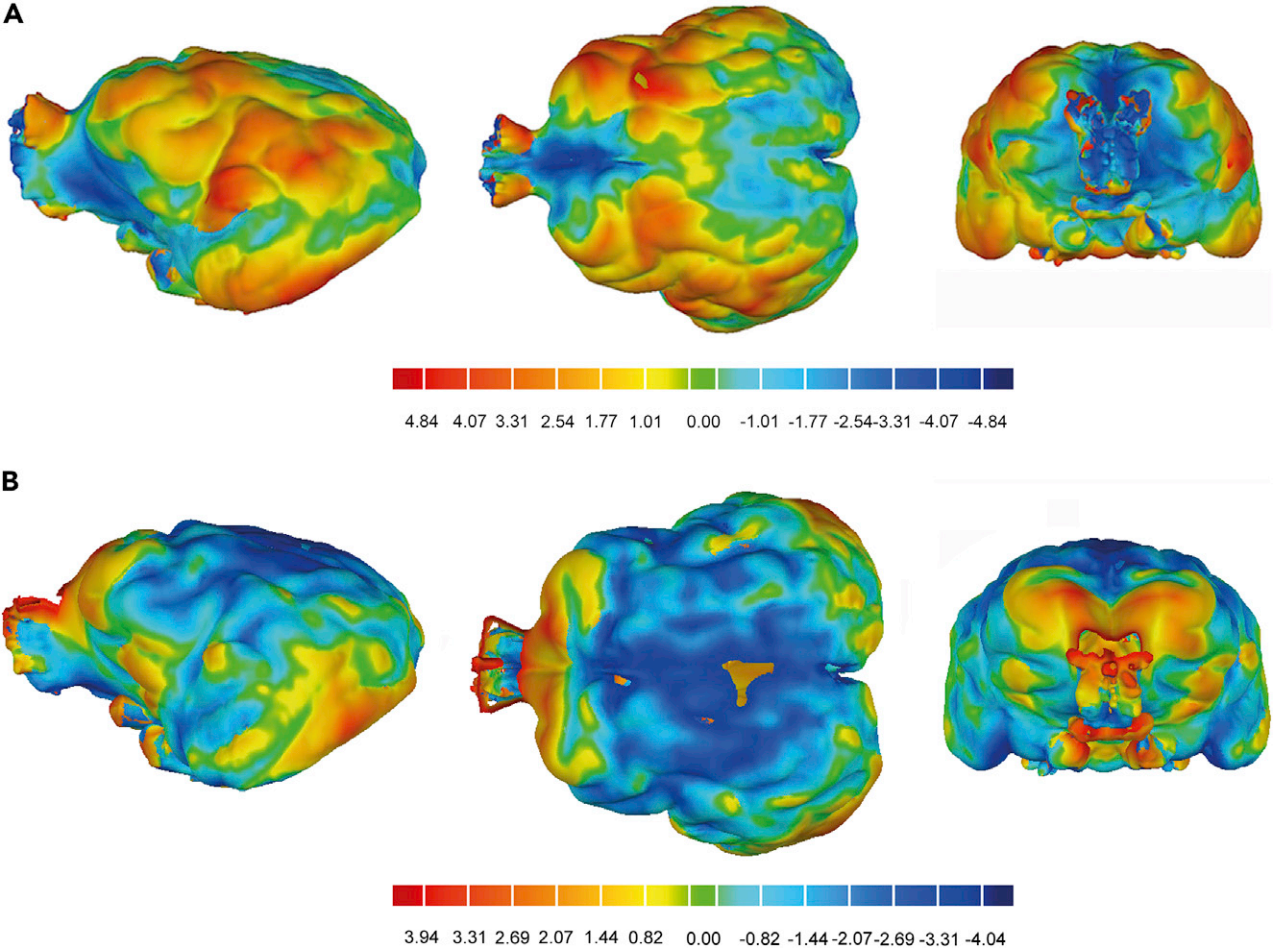
Topological deviations to the brain of *M. trumani* from the ones of *A. jubatus* and *P. concolor* (A) Deviations from *P. concolor* (reference) to *M. trumani* (target). (B) Deviations from *A. jubatus* (reference) to *M. trumani* (target). In both cases, warm colors indicate positive deviations (in mm) from the reference to *M. trumani*, and cold colors indicate negative deviations. See also Figure S2.

### Topological deviations from the brain of *M. trumani* to the ones of *A. jubatus* and *P. concolor*

The brain of *A. jubatus* has been proposed to be highly globose^55^ and our topological analysis confirms this. Comparing the brain topology of *P. concolor* with that of *M. trumani*, the latter have much more developed lateral sides, which may relate to a greater amplification of the motor, somatosensory, auditory, and visual regions of the cortex (Figure 3A)*.* On the other hand, the brain topology of *M. trumani* is closer (in terms of average distance) to that of *P. concolor* (0.23 ± 1.76 mm of average distance) than to that of *A. jubatus* (0.48 ± 1.66 mm of average distance), which probably relates to the more pronounced rostral dorsiflexion of the telencephalon in *A. jubatus* (Figure 3B). Following the areas defined in the cat brain,^58^ the areas more developed in *M. trumani* relative to *A. jubatus* are the prefrontal cortex (PFdm, PFdl), the motor cortex (4γ, 4δ, 6aα, 6aβ), the primary somatosensory cortex (1, 2, 3a, 3b), the fourth somatosensory (S4) and the partially fifth somatosensory cortex (S5), the visual cortex (20a, 21b, 17 and partially 21a, 19) and other areas of the auditory cortex (A2, dPE, iPE, vPE, VAF).

### Total and regional brain size in *M. trumani*

**Figure 4:**
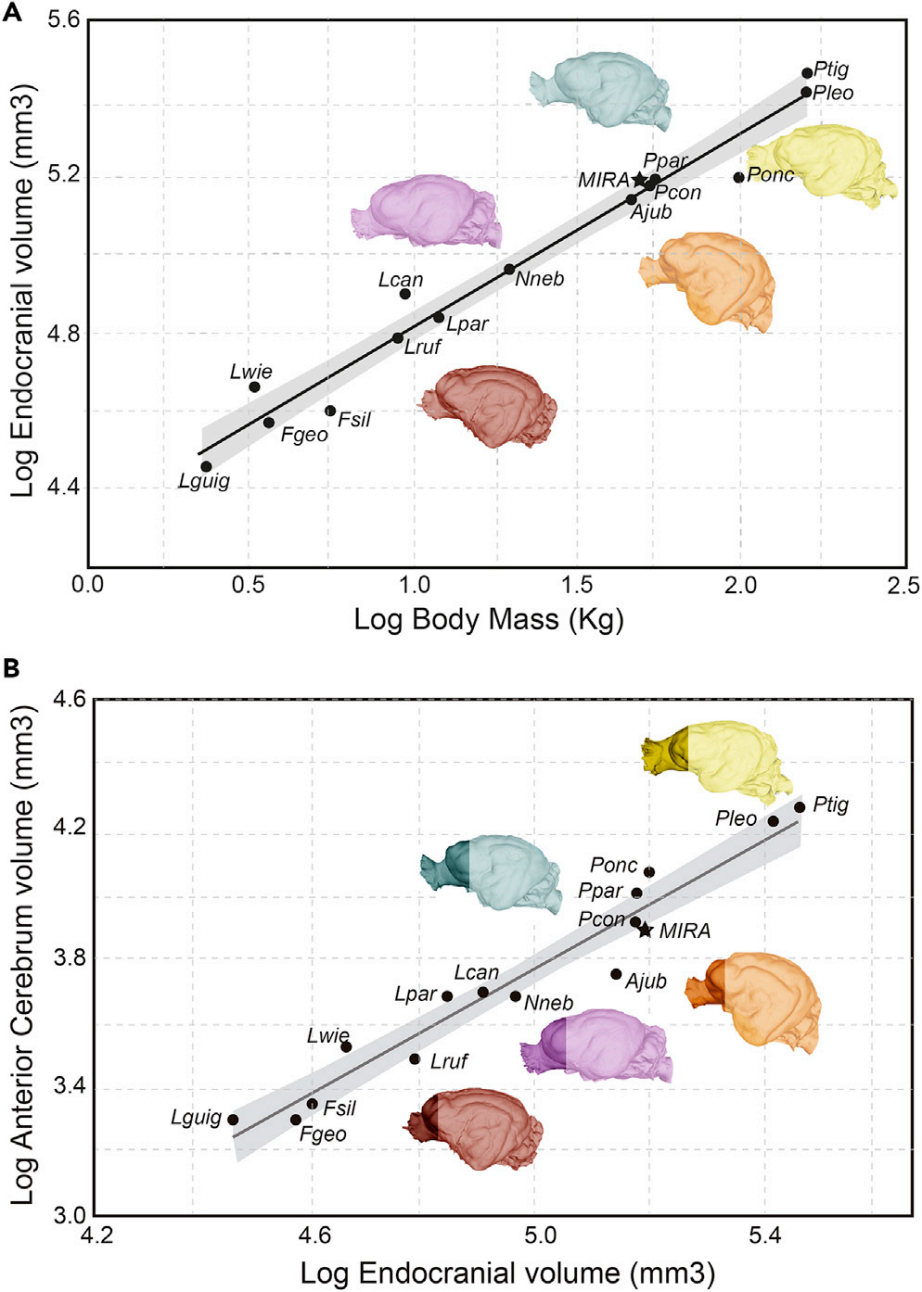
Total and regional brain size in *M. trumani* (A) Total endocranial volume (in mm^3^) against body mass (in kg), both variables log-transformed. (B) Anterior cerebrum volume (in mm^3^) against total endocranial volume (in mm^3^), both variables log-transformed. Abbreviations: Lguig, *Leopardus guigna*; Lwie, *Leopardus wideii*; Fgeo, *Felis geoffroyi*; Fsil, *Felis silvestris*; Lruf, *Lynx rufus*; Lpar, *Leopardus pardalis*; Lcan, *Lynx canadensis*; Nneb, *Neofelis nebulosa*; Ajub, *Acinonyx jubatus*; Ppar, *Panthera pardus*; Ponc, *Panthera onca*; Pcon, *Puma concolor*; Mira, *Miracinonyx trumani*; Ptig, *Panthera tigris*; Pleo, *Panthera leo*.

The bivariate regression of Total Endocranial volume (TEv) on body mass (BM) was significant (*r*^2^= 0.9756; *F*_(1,13)_= 520.18; p<0.0001; Table 1) as well as the regression of both contrasted variables (*r*^2^= 0.8828; *F*_(1,12)_= 90.36; p<0.0001). The TEv values for *P. concolor* as well as those of *M. trumani* fall closer to the regression line –i.e., within the 95% confidence interval (Figure 4A). On the other hand, our results indicate that the jaguar (*Panthera onca*) is the less encephalized large cat among the sample (Figure 4A).

**Table 1:** Regional brain volumes and body masses of extant felids used in this study

Species	Mass(kg)	TEv(mm^3^)	ACv(mm^3^)	PCv(mm^3^)	CBv(mm^3^)
*A. jubatus*	46.7	136,747.818	5687.272	101,852.738	27,644.508
*F. silvestris*	5.53	39,346.93	2268.24	28,035.93	8268.72
*L. geoffroyi*	3.59	36,842.94	2026.78	25,939.62	8185.28
*L. guigna*	2.23	28,505.6	2028.27	19,540.5	6258.3
*L. pardalis*	11.9	68,282.95	4866.14	46,593.1	14,905.56
*L.wiedii*	3.25	45,526.52	3371.04	31,506.63	9745.92
*L.canadensis*	9.37	78,873.34	4999.91	56,902.51	15,459.24
*L. rufus*	8.91	60,584.94	3145.84	43,061.71	12,817.8
*M. trumani*	50	152,756.41	7876.76	109,221.88	33,198.75
*N. nebulosa*	19.676	90,903.89	4861.54	63,650.09	20,728.67
*P. leo*	161.5	259,987.7567	16,490.56667	183,225.81	53,714.98
*P. onca*	100	156,479.09	11,540.05	108,563.6	32,399.69
*P. pardus*	52.038	149,319.62	9954.66	105,203.34	30,425.05
*P. tigris*	162.56	289,463.72	18,206.34	211,268.89	53,660.62
*P. concolor*	51.6	147,947.375	7980.15	106,105.9275	31,254.2

Abbreviations: TEv, total endocranial volume; ACv, anterior cerebrum volume; PCv, posterior cerebrum volume; CBv, cerebellum/brain stem volume. Body masses obtained from the literature.^59^^,^^60^ The body mass of *M. trumani* was obtained in this study from the regression of mass against skull length for modern felids.^61^ The data represent species averages computed from this study and from previous data. See method details and quantification and statistical analysis.^55^

The bivariate regression of the Anterior Cerebrum volume (ACv) against TEv shows a significant association (*r*^2^= 0.9485; *F*_(1,13)_= 239.5; p<0.0001; Table 1), even when taking into account the phylogenetic relationships of the species (*r*^2^= 0.9187; *F*_(1,12)_= 135.7; p<0.0001). Strikingly, although the ACv of *P. concolor* is not reduced relative to its TEv compared to other felids –it falls within the 95% confidence interval of the regression line– the ACv of *M. trumani* is slightly reduced to its estimated TEv, falling outside the 95% confidence interval below the regression line (Figure 4B). Despite this, the value of ACv for *M. trumani* is closer to the one of *P. concolor* than to the one of *A. jubatus* (Figure 4B).

**Figure 5:**
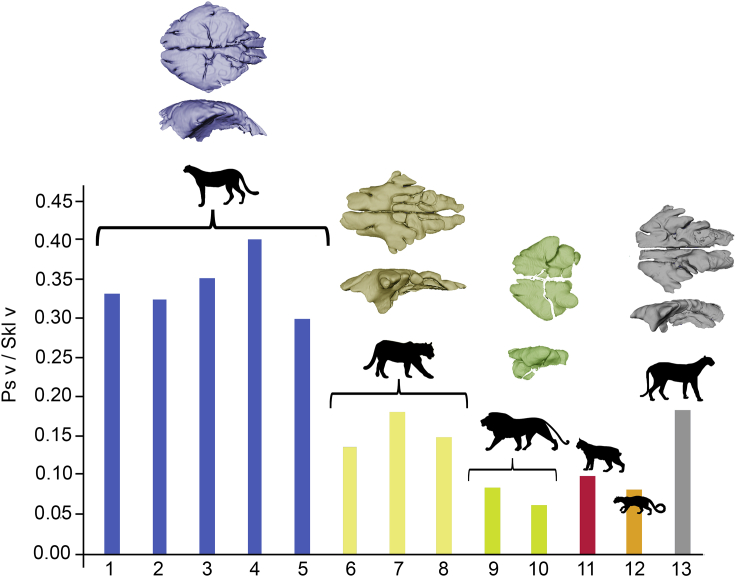
Paranasal sinuses size in *M. trumani* Histogram showing the ratio between paranasal sinuses volume and cranium volume. 1-5: *A. jubatus*; 6-8: *P. concolor*; 9,10: *P. leo*; 11: *L. rufus*; 12: *N. nebulosa*; 13: *M. trumani*. Abbreviations: Ps v, paranasal sinuses volume; Skl v, skull (cranium) volume. Paranasal 3D models are not scaled. Silhouettes obtained from Phylopic (phylopic.org).

### Paranasal sinuses size in *M. trumani*

The relative paranasal sinuses volume to total cranium volume is shown in Figure 5. Our results indicate that all specimens of *A. jubatus* possess a paranasal sinuses volume surpassing the 25% of cranium volume. In contrast, the paranasal sinuses volume of *M. trumani* and *P. concolor* varies between 15 and 20% of total cranium volume (Figure 5).

### General brain shape in *M. trumani*

**Figure 6:**
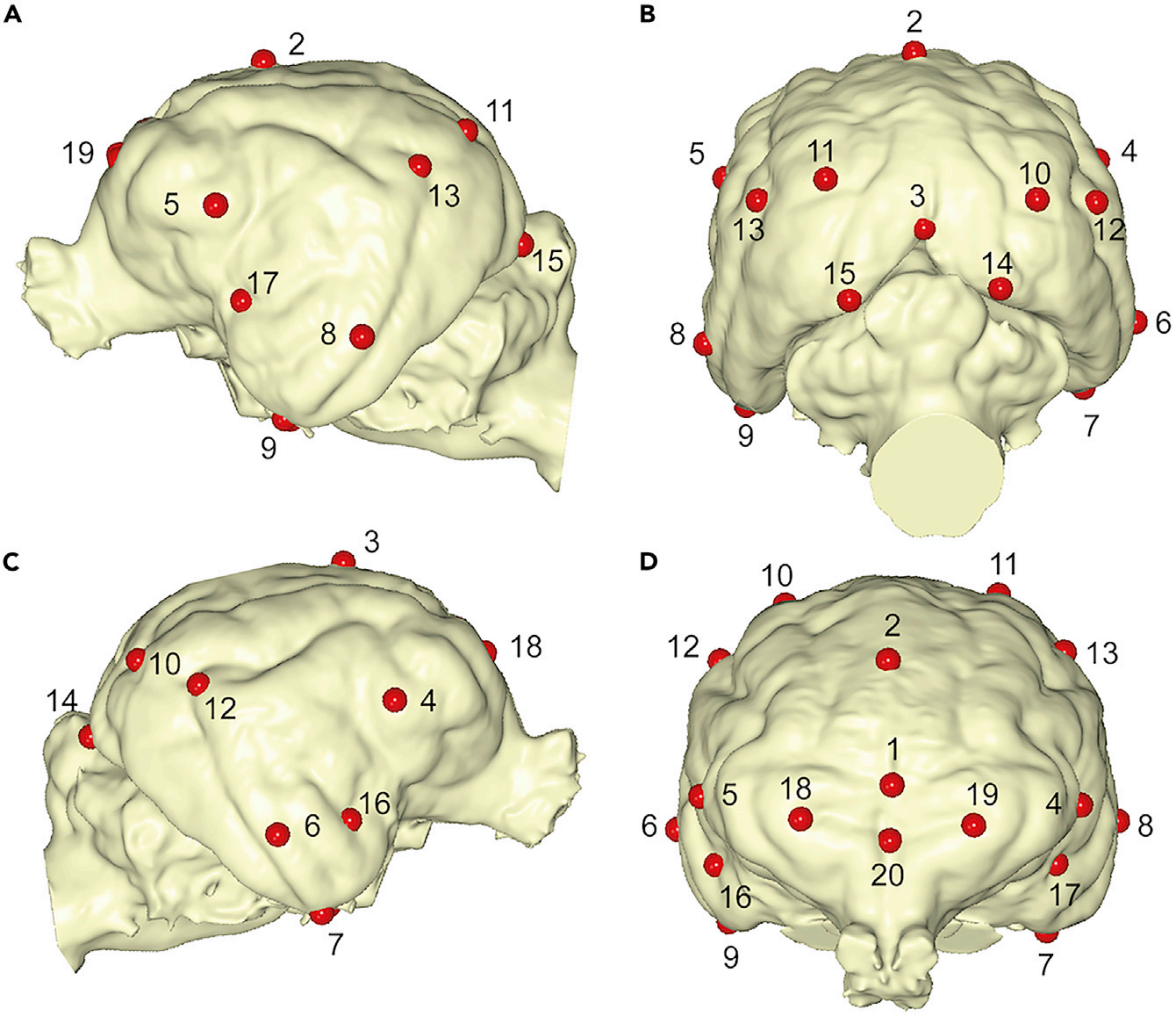
Landmarks digitized from brain endocasts for geometric morphometric analyses (A) Left lateral view. (B) posterior view. (C) right lateral view. (D) frontal view. Landmarks are defined as follows: 1: mid-sagittal point in frontal location; 2: mid-sagittal point in dorsal location; 3: mid-sagittal in caudal location; 4: most-lateral point of right frontal lobe; 5: most-lateral point of left frontal lobe; 6: most-lateral point of right temporal lobe; 7: lowest point of right temporal lobe; 8: most-lateral point of left temporal lobe; 9: lowest point of the left temporal lobe; 10: point of maximum curvature of the right suprasylvian gyrus; 11: point of maximum curvature of the left suprasylvian gyrus; 12: point of maximum curvature of the right ectosylvian gyrus; 13: point of maximum curvature of left ectosylvian gyrus; 14: most-posterior point of the right occipital gyrus; 15: most-posterior point of the left occipital gyrus; 16: deepest point of the right sylvian sulcus; 17: deepest point of the left sylvian sulcus; 18; most-lateral point of the right cruciate sulcus; 19: most-lateral point of the left cruciate sulcus; 20: midpoint of the cruciate sulcus.

**Figure 7:**
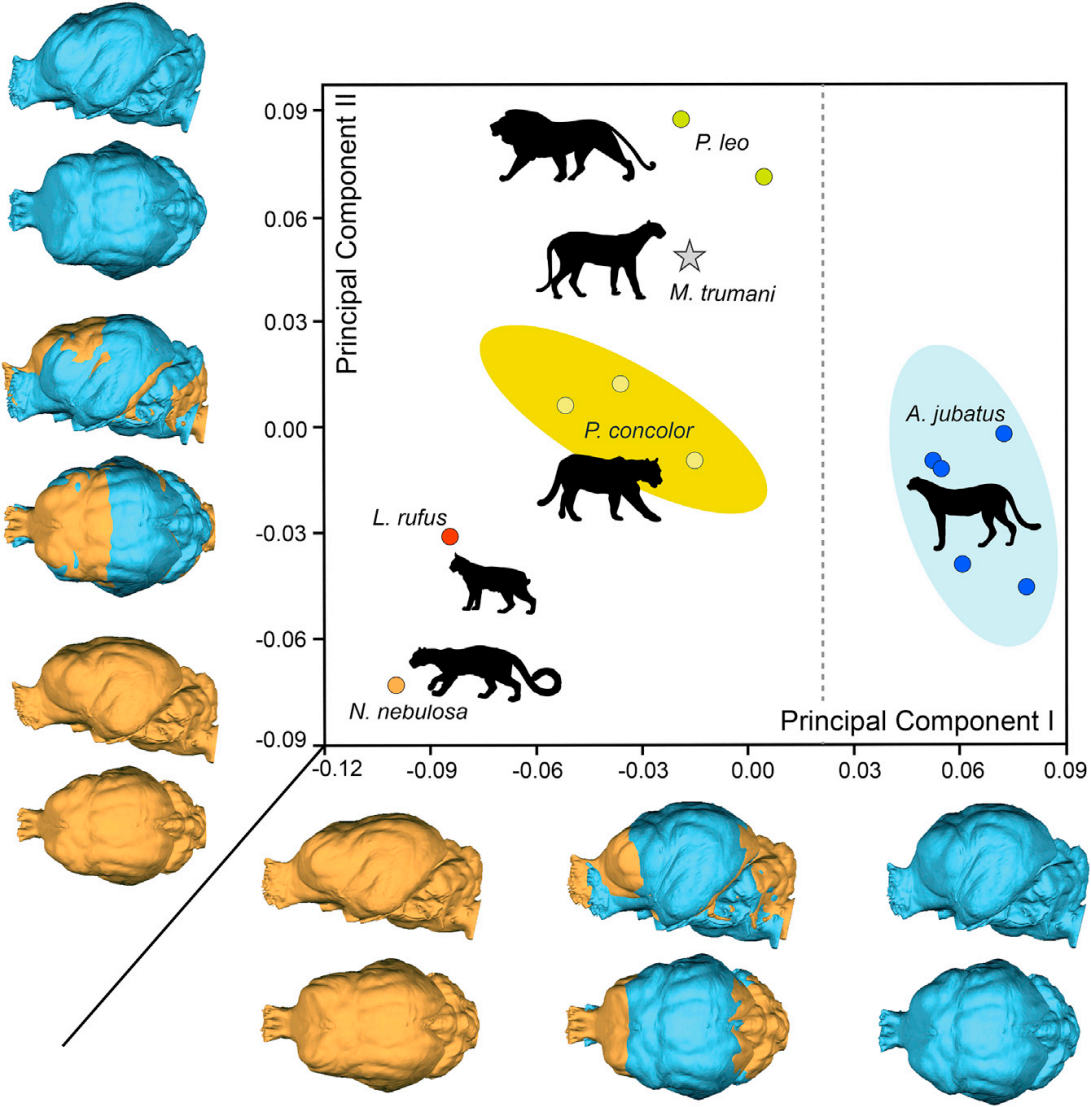
General brain shape in *M. trumani* Morphospace depicted by the first two PCs obtained from a Principal Component Analysis of 20 landmarks digitized in 13 brain endocasts. Silhouettes obtained from Phylopic (phylopic.org). See also Figure 6.

The bivariate plot depicted by the first two eigenvectors obtained from a PCA of the 20 landmarks digitized (Figure 6) on the brain endocasts is shown in Figure 7. Inspection of other PCs do not reveal a clear pattern of specimen ordination, as they highlight few taxa. The first PC, which explains 34.35% of the original variance, mainly separates *A. jubatus* from the other felids. The morphological variation accounted for by this eigenvector is mainly related to the antero-posterior location of the most-lateral point of the frontal lobe (i.e, *landmarks* 4,5) and the mid-sagittal brain (i.e, *landmarks* 2) (Figure 7).

The second PC, which explains 20.36% of the original variance, mainly separates among the felids *P. leo* and *M. trumani*, which both take positive scores, from *P. concolor*, which score with intermediate values, and *Neofelis nebulosa* and *Lynx rufus*, which both take negative projections (Figure 7). The morphological variation accounted for by this eigenvector relates to the antero-posterior location of the cruciate sulcus (i.e., *landmarks* 18,19), the most-lateral (i.e., *landmarks* 6,7) and lowest (i.e., *landmarks* 8,9) points of the temporal lobe, as well as the maximum curvature of the right and left suprasylvian gyrus (i.e., *landmarks* 10,11) (Figure 7).

## Discussion

Our results indicate that, compared to *A. jubatus*, the suprasylvian sulcus is not disrupted in either *M. trumani* or *P. concolor*, although the continuation of this sulcus in *M. trumani* is subtle. Strikingly, this disruption has been interpreted as a consequence of the expansion of the Clare-Bishop area, which probably relates to a visual specialization in *A. jubatus,* a diurnal predator that relies on eyesight for prey detection and chase.^57^ Our results for *M. trumani* may tentatively indicate that it also relied on eyesight for detecting prey, but to a lesser degree than the cheetah. Moreover, the orbital sulcus of *M. trumani* does slightly continue with the ectosylvian sulcus, a pattern that is different from the one observed in *A. jubatus*. The interruption of orbital-ectosylvian sulci in the cheetah relates to its more globose brain, which in turn relates to the position of the anterior coronal and anterior suprasylvian gyri that bulge out more beyond the lateral boundary of the sigmoid gyri than in other similar-sized felids.^57^ Therefore, the continuation of the orbital-ectosylvian sulci in *M. trumani* reflects that its brain is more globose than the one of *P. concolor,* although it does not reach the extreme degree observed in *A. jubatus.* This is also confirmed by our topological analysis of the brain of *M. trumani*. However, our results indicate that compared to the brain of *P. concolor,* the brain of *M. trumani* is characterized by the presence of a well-developed somatosensory cortex and visual areas, as well as by the expansion of other areas related to the auditory cortex. Although this may indicate that *M. trumani* has enhanced visual and auditory functions compared to *P. concolor*, it also exhibits a greater motor complexity than *A. jubatus*, the latter probably related to the presence of fully retractable claws in *M. trumani*.^43^ In any case, future quantitative studies of brain functional areas will clarify which brain functions in *M. trumani* are enhanced or diminished compared to both *Puma* and *Acinonyx*.

Following a previous study,^57^ the postcruciate sulcus separates the primary motor and somatosensory cortical areas that control the postcranial part of the body. The lack of that sulcus in *A. jubatus* has been interpreted as reflecting its relatively small motor cortex, which would be related to the presence of a less developed limb musculature.^55^^,^^57^ The degree of forepaw dexterity in *A. jubatus* ranks the lowest among the modern felids,^18^ mainly because of its reduced ability to supinate the forelimb.^2^^,^^19^^,^^20^^,^^21^^,^^62^ As a result, its ability to climb trees and manipulate prey is diminished compared to other taxa.^63^ This likely relates to its specialized predatory behavior, which is based in a fast-running chase at the expense of the loss of manipulatory capabilities with the forelimb.^14^^,^^64^ Unlike other felids, *A. jubatus* lacks fully retractable claws, which improves limb traction and support.^14^ Most cats use their claw-equipped forelimbs to grapple and manipulate prey. This is especially relevant for large-sized species, which typically take prey with a body size that equals to, or is greater than, their own.^65^*A. jubatus* is the only large cat that regularly kills prey with a body weight that is less than its own^16^^,^^66^^,^^67^ and its killing bite is performed at the ventral region of the neck by strangulation, bringing down prey by hooking one of its dewclaws into the animal and shifting its own weight posteriorly.^14^^,^^22^^,^^68^^,^^69^ Indeed, it has been proposed that although the dewclaw in the tiger (*P. tigris*), lion (*P. leo*) and leopard (*Panthera pardus*) is only slightly larger than the claw of the second digit, the dewclaw of the cheetah is enlarged relative to other digits, a condition that should be related to its separate role.^23^ On the other hand, the dewclaw of *P. concolor* was intermediate in size to that of pantherines and that of *A. jubatus*, and Londei^23^ explained the condition of *P. concolor* as a leftover trait that was probably inherited from more cursorial (extinct) forms. However, although the size of the dewclaw could be a key trait for the interpretation of the predatory behavior of *M. trumani,* whether digit I is also enlarged in this species remains to be investigated.

It is worth noting that the postcruciate sulcus is also present in the canids, which do not possess retractable claws (excepting *Urocyon cinereoargenteus*) and use their forelimbs almost exclusively for running.^2^^,^^20^^,^^64^ However, the postcruciate sulcus appeared independently in canids and felids.^70^

It is surprising that even though *M. trumani* is extremely cheetah-like in appearance and limb proportions, it shows fully-retractable claws. This could explain the presence in this predator of an expanded motor cortex compared to *A. jubatus.* In turn, this could relate to its greater ability for prey manipulation and support, which could explain the retention of *M. trumani* (as in *P. concolor*) of the postcruciate sulcus as a small dimple. However, future ecomorphological studies of *M. trumani* based on its major limb bones could give some clues on this topic, as ecomorphological studies performed on the appendicular skeleton has provided proxies of predatory behavior in the living carnivorans.^2^^,^^13^^,^^17^^,^^20^^,^^21^^,^^71^^,^^72^^,^^73^^,^^74^^,^^75^^,^^76^^,^^77^

On the other hand, the brain of *A. jubatus* is the smallest relative to its body mass among the living felids,^55^ which has been interpreted as an adaptation for weight loss and energy saving, aspects that might be advantageous for a predatory behavior based on a fast-running chase. If this explanation holds true, the endocranial volume for *M. trumani* could tentatively indicate that this predator was not as equipped for fast-running as *A. jubatus*. However, our data suggests that the TEv of *A. jubatus* is not more reduced than that of other felids such as the jaguar (*P. onca*), which is a generalized predator.^59^ However, it is worth noting that the body mass of *P. onca* is proportionately elevated, rather than having reduced TEv relative to body mass.

Strikingly, the volume of the anterior cerebrum of *A. jubatus*, which apparently relates to the degree of sociality in carnivores, is highly reduced compared to other felids^55^*–*even though males can form coalitions of 2 or 3 related individuals,^78^^,^^79^ they do not cooperate during a hunt.^80^ However, it is worth noting that the relatively small brain of *A. jubatus* could be also explained by its low genetic diversity because of past population bottlenecks,^81^^,^^82^^,^^83^ although we find this possibility speculative. In any case, the anterior cerebrum of *M. trumani* is also reduced compared to *P. concolor*, but without reaching the extreme reduction seen in *A. jubatus*.

Apart from these differences, the brain of *A. jubatus* also differs from the other felids in general shape. For example, it has been noted that the brain of the cheetah shows a unique rostral dorsiflexion among felids.^55^ Our geometric morphometric analysis, based on 3D landmarks digitized from relative positions of gyri and sulci, indicates that the brain of *A. jubatus* possesses more posteriorly positioned frontal lobes and more anteriorly positioned mid-sagittal brains than in other felids. This overally relates to the brain dorsiflexion of the cheetah.^55^ In this respect, the brain of *M. trumani* does not significantly differ from that of other felids, and therefore, it is not characterized by having the dorsiflexion typical of *A. jubatus*.

Although, fitting the brain into the bony skull with its functions requires integration between the two parts^84^^,^^85^^,^^86^ and the ‘spatial packing hypothesis’ points that spatial constraints might shape brains to be packed more globularly when their mass increases relative to body mass,^87^ the overall cranium shape of *Acinonyx* and *M. trumani* is very similar and rather distinct of other felid species.^52^ Therefore, the influence of external cranium shape on endocast form could be in principle negligible. This could be extended to cranium function (e.g., feeding) because the latter is reflected on cranium shape in carnivores.^88^^,^^89^^,^^90^^,^^91^^,^^92^ However, this rostral dorsiflexion of the brain of *A. jubatus* reflects the presence of enlarged frontal sinuses,^25^ which leads to its highly domed cranium.^28^ The enlargement of the frontal sinuses is thought to act as a vascular cooling mechanism during high-speed chases.^25^^,^^35^ Indeed, at the end of a sprint, the body temperature of *A. jubatus* is ∼41°C^6^^,^^10^ and the large frontal sinus plays an important role in preventing brain overheating.^25^ Again, our qualitative assessment of paranasal sinuses volume in *M. truman*i indicates that it did not possess the enlarged sinuses typical of *A. jubatus.* Therefore, brain overheating was probably not a selective agent here. This could be related to the fact that *M. trumani* did not perform the bursts of speed of a cheetah, or could even reflect that Pleistocene temperatures in North America were much lower than today in Africa and Iran.^93^^,^^94^ In any case, the absence of well-developed sinuses in *M. trumani* is probably behind the lack in this felid of the brain rostral dorsiflexion typical of *A. jubatus*.

In summary, all these morphological traits seem to indicate that *M. trumani* was not as specialized as *A. jubatus* in deploying a fast-running pursuit or, at least, that it was not ‘cognitively’ equipped for this predatory behavior. Therefore, given the skeletal resemblance between *M. trumani* and *A. jubatus*, it is reasonable to think that the skeletal adaptations for fast-running evolved faster or earlier than those related with the architecture of the brain. However, our results also indicate that *M. trumani* does not possess paranasal sinuses as developed as the living cheetah –although this trait has been traditionally used to justify the convergent evolution between *M. trumani* and *A. jubatus*.^44^^,^^45^ This evidence, together with the presence of fully-retractable claws in *M. trumani,* cast doubts that *M. trumani* deployed a fast-running chase as specialized as in *A. jubatus*. However, although the absence of fully-retractable claws has been interpreted as an adaptation to enhance limb traction and support during a chase,^14^ this interpretation could be biased for the absence of retractable claws in the pack-hunting canids. To us, it is not really clear whether *A. jubatus* lost its fully retractable claws as a response of its highly specialized predatory behavior based in fast-pursuit of open-country prey, or it was just a consequence of preying upon small prey. If the second possibility holds, the presence of retractable claws in *M. trumani* would not be an argument to support the hypothesis that it was not as specialized as *A. jubatus* for fast running. Instead, it would indicate that it preyed upon larger prey.

On the other hand, it has been found remains of *M. trumani* in different caves from the Grand Canyon of northern Arizona and proposed that *M. trumani* should be envisaged as a species better adapted to dry uplands and rocky canyons, and not restricted to savanna-like settings.^95^ In this respect, it is claimed that the ecology of the Grand Canyon *M. trumani* was similar to the living Asiatic cheetah (*Acinonyx jubatus venaticus*) and snow leopard (*Panthera uncia*), which are adapted for pursuit of mountain and canyon ungulates over near vertical rocky and mountainous terrain.^95^

In any case, our results were totally unexpected given the overall skeletal (and dental) resemblance between *A. jubatus* and *M. trumani,* but ecomorphological studies from limb bones in *M. trumani* could answer whether the skeleton of this predator was already equipped for a fast-running chase and whether skeletal modification preceded the evolution of brain architecture in this formidable lineage of big cats.

### Limitations of the study

Our study is based on a single endocast virtually extracted from a cranium preserved in Natural Trap Cave. As a result, we lack the broader perspective offered by large intraspecific studies. Moreover, following previous studies^57^ we assumed that the distinctive traits of *A. jubatus* brain are related to its predatory behavior, but this could also relate to its genetic impoverishment because of the severe population bottlenecks experienced by this species in the past.

Another caveat we find in our study is the possibility that the brain of *A. jubatus* could be (in part) the result of its expanded sinuses. Future studies investigating the role of sinus development in *A. jubatus* to explain the characteristic dorsiflexion of its brain are necessary to confirm or refute whether the brain architecture of *A. jubatus* is partially a by-product of other internal structures such as the sinuses.

## STAR★Methods

### Key resources table

**Table:** 

REAGENT or RESOURCE	SOURCE	IDENTIFIER
**Biological samples**		

*Miracinonyx trumani*	University of Kansas Natural History Museum	KUVP-51277
*Acinonyx jubatus*	University of Wisconsin Zoological Collection	UWZS 23961
*Acinonyx jubatus*	Field Museum of Natural History (Chicago, USA)	FMNH 29635
*Acinonyx jubatus*	Field Museum of Natural History (Chicago, USA)	FMNH 127834
*Acinonyx jubatus*	Osteological Museum of Valladolid University	VU 6075
*Acinonyx jubatus*	Osteological Museum of Valladolid University	VU 6394
*Puma concolor*	University of Wisconsin Zoological Collection	UWZS 32281
*Puma concolor*	Osteological Museum of Valladolid University	VU 409
*Puma concolor*	Osteological Museum of Valladolid University	VU 3087
*Panthera leo*	Osteological Museum of Valladolid University	VU 6080
*Panthera leo*	Osteological Museum of Valladolid University	VU 2685
*Lynx rufus*	Ohio University Vertebrate Collection	OUVC 9576
*Neofelis nebulosa*	National Museum of Natural History, Washington DC (USA)	USNM 282124

**Deposited data**		

Endocast models	Figshare	Data from: Built for speed? The brain of the North American cheetah-like cat *Miracinonyx trumani*, https://figshare.com/s/b1366f7e7aef6317c62e
Scanning parameters	supplemental information	Table S1

**Software and algorithms**		

MorphoJ	Klingenberg^96^	https://morphometrics.uk/MorphoJ_page.html
Rstudio	R core team^97^	https://www.rstudio.com/
3D-slicer	Kikinis et al.^98^	https://www.slicer.org/
Fiji (ImageJ)	Schindelin^99^	https://imagej.net/software/fiji/
Geomagic studio	3D Systems^100^	https://es.3dsystems.com/software
Geiger package	Harmon et al.^101^	https://cran.r-project.org/web/packages/geiger/index.html
Mesquite	Maddison and Maddison^102^	https://www.mesquiteproject.org/
Ape	Paradis et al.^103^	https://cran.r-project.org/web/packages/ape/index.html

### Resource availability

#### Lead contact

Further information and requests for resources should be directed to and will be fulfilled by the lead contact, Dr. Borja Figueirido (Borja.figueirido@uma.es).

#### Materials availability

• This study did not generate unique reagents.

### Experimental model and subject details

#### CT-scanning of data

We CT scanned a complete cranium of *M. trumani* (KUVP-51277) unearthed from Natural Trap Cave (northern Wyoming, USA) with an age of *ca*. 23-25 ka^96^. Additional skulls of other felid species were also CT-scanned: 5 cheetahs (*A. jubatus*), 2 lions (*Panthera leo*), 3 cougars (*Puma concolor*), 1 clouded leopard (*Neofelis nebulosa*), and 1 bobcat (*Lynx rufus*). See Table S1.

Cranium KUVP-51277 of *Miracinonyx trumani* was unearthed from Natural Trap Cave (northern Wyoming, USA) with an age of *ca*. 23-25 ka, Late Pleistocene^104^ and it is housed in the University of Kansas Vertebrate Paleontology (KUVP). The cranium of this specimen was CT-scanned at University at the Wisconsin Institute for Medical Research’s Imaging Services Department of the University of Wisconsin with a GE Medical System Discovery model CT750 in Helicoidal mode. The acquisition parameters were: Kvp 120; X-ray tube current 250A (Ampere); slice thickness 0.625; spacing between slices 0.3120; exposure time 912; field of reconstruction 143 mm; pixel spacing 0.2793 mm; pixel size 512x512 and voxel size 0.2793 X 0.2793 X 0.3120 mm. Number of images in set 2974 in 16-bit TIFF images. See Table S1.

Cranium UWZS 23961 of *Acinonyx jubatus* belongs to a captive female from William Lowe Game Farm, Beaver Dam, Dodge County, Wisconsin, USA. This specimen was scanned at the Wisconsin Institute for Medical Research’s Imaging Services Department (University of Wisconsin). The CT scan machine used is a GE Medical System Discovery CT750. The CT scanner was done in Helicoidal mode. The acquisition conditions were as follows: Kvp 120; X-ray tube current 250 A (Ampere); slice thickness 0.625; spacing between slices 0.3122; exposure time 912; field of reconstruction 222 mm; pixel spacing 0.4336 mm; pixel size 512x512 and voxel size 0.4336 X 0.4336 X 0.3122 mm. Number of images in set 1848 in 16-bit TIFF images. See Table S1.

Cranium FMNH 29635 of *Acinonyx jubatus raineyii* is a male of wild origin from Eastern Kenya housed in the Field Museum of Natural History of Chicago (USA) and it was CT-scanned at the University of Texas High-Resolution X-ray CT-Facility using the following parameters: Kvp 420; X-ray tube current 180 A (Ampere); slice thickness 0.5 mm; inter slices 0.48 mm; field of reconstruction 131 mm; pixel spacing 0.26 mm; pixel size 512x512 and voxel size 0.26 X 0.26 X 0.48 mm. Number of images in set 393 in 16-bit TIFF images. See Table S1.

Cranium FMNH 127834 of *Acinonyx jubatus raineyii* belongs to a female of wild origin from Kenya and it is housed in the Field Museum of Natural History (Chicago, USA). This specimen was scanned at the University of Texas High-Resolution X-ray CT Facility. The acquisition conditions were: Kvp 420; X-ray tube current 180 A (Ampere); slice thickness 0.5 mm; inter slices 0.46 mm; field of reconstruction 131 mm; pixel spacing 0.25 mm; pixel size 507x402 and voxel size 0,25 X 0.25 X 0.46 mm. Number of images in set 363 in 16-bit TIFF images. See Table S1.

Cranium VU 6075 of *Acinonyx jubatus* belongs to a captive male and it is housed in the Osteological Museum of Valladolid University. Such specimen was scanned at Vithas Medical Center (Malaga, Spain). The skull was CT-scanned with a GE Medical Systems (Brivo CT385 Series) and the conditions of acquisition were: Kvp 120; X-ray tube current 95 A (Ampere); slice thickness 0.625mm; inter slices 0.31 mm; field of reconstruction 265 mm; pixel spacing 0.517 mm; pixel size 512x512 and voxel size 0.517 X 0.517 X 0.31 mm. Number of images in set 1234 in 16-bit TIFF images. See Table S1.

Cranium VU 6394 of *Acinonyx jubatus* belongs to a captive female and it is housed in the Osteological Museum of Valladolid University. Such specimen was scanned at Vithas Medical Center (Malaga, Spain). The skull was CT-scanned with a GE Medical Systems (Brivo CT385 Series) and the conditions of acquisition were: Kvp 120; X-ray tube current 95 A (Ampere); slice thickness 0.625mm; inter slices 0.31 mm; field of reconstruction 265 mm; pixel spacing 0.517 mm; pixel size 512x512 and voxel size 0.517 X 0.517 X 0.31 mm. Number of images in set 1234 in 16-bit TIFF images. See Table S1.

Cranium UWZS 32281 of *Puma concolor* belongs to a wild animal from Wyoming, USA. This specimen was scanned at the Wisconsin Institute for Medical Research’s Imaging Services Department (University of Wisconsin). The CT scan machine used is a GE Medical System Discovery CT750. The CT scanner was done in Helicoidal mode. The acquisition conditions were: Kvp 120, X-ray tube current 250 A (Ampere), slice thickness 0.625, spacing between slices 0.3122, exposure time 912, field of reconstruction 179 mm; pixel spacing 0.3496 mm, pixel size 512x512 and voxel size 0.3496 X 0.3496 X 0.3122 mm. Number of images in set 2862 in 16-bit TIFF images. See Table S1.

Cranium VU 409 of *Puma concolor* belongs to a captive male and it is housed at the Osteological Museum of Valladolid University. Such specimen was scanned at Vithas Medical Center (Malaga, Spain). The skull was CT-scanned with a GE Medical Systems (Brivo CT385 Series) and the conditions of acquisition were: Kvp 120; X-ray tube current 114 A (Ampere); slice thickness 0.625mm; inter slices 0.31 mm; field of reconstruction 250 mm; pixel spacing 0.4883 mm; pixel size 512x512 and voxel size 0.4883 X 0.4883 X 0.31 mm. Number of images in set 1040 in 16-bit TIFF images. See Table S1.

Cranium VU 3087 of *Puma concolor* belongs to a captive female and it is housed in the Osteological Museum of Valladolid University. Such specimen was scanned at Vithas Medical Center (Malaga, Spain). The skull was CT-scanned with a GE Medical Systems (Brivo CT385 Series) and the conditions of acquisition were: Kvp 120; X-ray tube current 80 A (Ampere); slice thickness 0.625mm; inter slices 0.31 mm; field of reconstruction 367 mm; pixel spacing 0.7168 mm; pixel size 512x512 and voxel size 0.7168 X 0.7168 X 0.31 mm. Number of images in set 1195 in 16-bit TIFF images. See Table S1.

Cranium VU 6080 of *Panthera leo* belongs to a captive male and it is housed in the Osteological Museum of Valladolid University. Such specimen was scanned at Vithas Medical Center (Malaga, Spain). The skull was CT-scanned with a GE Medical Systems (Brivo CT385 Series) and the conditions of acquisition were: Kvp 120; X-ray tube current 106 A (Ampere); slice thickness 0.625mm; inter slices 0.31 mm; field of reconstruction 319 mm; pixel spacing 0.6231 mm; pixel size 512x512 and voxel size 0.6231 X 0.6231 X 0.31 mm. Number of images in set 1161 in 16-bit TIFF images. See Table S1.

Cranium VU 2685 of *Panthera leo* belongs to a captive female housed in the Osteological Museum of Valladolid University. Such specimen was scanned at Vithas Medical Center (Malaga, Spain). The skull was CT-scanned with a GE Medical Systems (Brivo CT385 Series) and the conditions of acquisition were: Kvp 120; X-ray tube current 75 A (Ampere); slice thickness 0.625mm; inter slices 0.31 mm; field of reconstruction 288 mm; pixel spacing 0.5625 mm; pixel size 512x512 and voxel size 0.5625 X 0,5625 X 0.31 mm. Number of images in set 1450 in 16-bit TIFF images. See Table S1.

Cranium OUVC 9576 of *Lynx rufus* belongs to a wild specimen and it is housed in the Ohio University Vertebrate Collection. The CT-scanner was a General Electric eXplore Locus *in vivo*, Ohio University MicroCT Facility (Ohio University). The acquisition conditions were: Kvp 80; X-ray tube current 450 A (Ampere); inter slices 0.09 mm; pixel spacing 0.09 mm; pixel size 1072x728 and voxel size 0.09 X 0.09 X 0.09 mm. Number of images in set 1580 in 16-bit TIFF images. See Table S1.

Cranium USNM 282124 of *Neofelis nebulosa* belongs to a captive male and it is housed in Smithsonian Institution, National Museum of Natural History, Washington DC (USA). This specimen was scanned at the University of Texas High-Resolution X-ray CT Facility. The acquisition conditions were: Kvp 419; X-ray tube current 180 A (Ampere); slice thickness 0.25 mm; inter-slices 0.25 mm; field of reconstruction 194 mm; pixel spacing 0.1890 mm; pixel size 1024x1024 and voxel size 0.1890 X 0.1890 X 0.25 mm. Number of images in set 353 in 16-bit TIFF images. See Table S1.

### Method details

#### Segmentation of brain endocasts

Data were saved as Digital Imaging and Communications in Medicine Centricity (DICOM) in 16 bits and imported into 3D-slicer^98^ to obtain 3D endocasts. An improvement of the definition of the internal structures and the topology of the brain endocast was performed using the resampling tool in ImageJ using Fiji^99^ following previous studies.^105^

Following previous studies,^55^ we selected the empty endocranial space in each coronal section from the cribriform plate to the opening of the foramen magnum. Afterwards, we compiled all slices to render a 3D virtual endocast. We also segmented the paranasal sinuses in our 12 specimens to calculate paranasal sinuses volume, in parallel with the volume of the brain endocast.

The extracted endocasts are available at Figshare (https://figshare.com/s/b1366f7e7aef6317c62e).

#### Investigation of gyri and sulci

To investigate the pattern of gyri and sulci in *M. trumani* from its brain endocast, we followed the terminology of previous authors^57^^,^^58^^,^^106^ as brain maps to identify those gyri and sulci in our sample, especially in *A. jubatus*, *M. trumani* and *P. concolor*. Moreover, as the gyri and sulci are not easily identifiable in those brain endocasts obtained from medical CTs because they can be blurred, we applied the curvature map in Geomagic Essentials,^100^ which quantifies the mean curvature (MC) as the average value between maximal and minimal curvatures in local surfaces (see Figure S1). The value of MC allows to classify the vertices and generate a color pattern based on concave (MC<0, blue), convex (MC>0, red) and flat (MC=0, green) surfaces.^105^^,^^97^

### Quantification and statistical analysis

We performed two topological analyses to compare quantitatively the endocast of *M. trumani* (target) with that of *P. concolor* and *A. jubatus*. To eliminate size effects in both comparisons, the target model was scaled to that of reference models for each comparison. The target endocast was increased by a scale factor of 5% (*M. trumani* vs. *P. concolor*), and reduced in size by a factor of 5% (*M. trumani* vs. *A. jubatus*) (Figure 4). We also quantified topological deviation between the target and reference models in each comparison using an alignment with the minimum average distance models with the software Geomagic essentials (https://es.3dsystems.com/software).See also Figures S1 and S2.

To do this, we fitted the two meshes for each comparison so that the distance among them should be the minimum. Accordingly, the target model is scaled to the reference model. This is necessary to perform a better pairwise superimpositions using the best fit algorithm during the alignment.

The alignment algorithm matches the coordinates of the target model to the reference model. The deviation between both geometric shapes is minimized within a tolerance of <1mm. This process uses the options of exhaustive symmetry and high-precision assembly algorithm, through a point-to-point resampling between the two topologies, choosing the closest points in each iterative process. Each inspection was carried out under a sampling relation of 100% and a maximum iteration count of 5000. The average positive and negative deviations, standard deviations, and the root-mean-square mean square values were obtained from each deviation output of Euclidean distance between the target and reference models.

From these data, we quantified the topological deviation between the target and reference models in each comparison using an alignment with the minimum average distance models previously explained (Figures S1 and S2). In the first topological analyses, we compared *M. trumani* with *P. concolor* (Figures S1 and S2), obtaining an average distance of 0.23 mm between the two models, a standard deviation of 1.75 mm and a root mean square of 1.177. In the second topological analyses, we compared the brain endocast of *M. trumani* with that of *A. jubatus* (Figures S1 and S2), obtaining an average distance of 0.48 mm, a standard deviation of 1.66 and a root mean square of 1.73 mm.

#### Quantifying regional and total brain sizes

We used osteological landmarks and gyral/sulcal patterns following previous authors^55^ to subdivide the endocast into four brain regions (olfactory bulb [OB], anterior cerebrum [AC], posterior cerebrum [PC], and cerebellum/brain stem [CB+BS], –i.e. cerebellum plus medulla, pons and part of the caudal mesencephalon) (Figure 1) to calculate regional volumes of these brain regions (OBv, ACv, PCv, CB+BSv). Total endocranial and regional volumes –as well as paranasal sinuses volume– were obtained using Geomagic Essentials^100^ and are shown in Table 1. Moreover, the calculated volumes of these regions were summed to obtain a total endocranial volume (TEv).

We regressed the total endocranium volume (TEv) against the body mass (BM) of each species (Table 1) to investigate whether *M. trumani* possessed an endocranial volume comparable to the one of *A. jubatus*. The BMs of all living felid species were taken from the literature.^60^ The body mass of *M. trumani* was calculated from the regression equation of skull length against body mass of modern felids.^61^ Skull length of *M. trumani* skull was measured with Geomagic Essentials^100^ from the 3D model and gave 17.8 cms. Moreover, given that other authors^55^ observed that the brain of *A. jubatus* is characterized by having a significantly lower ACv relative to TEv, we also regressed these variables. In both cases, we used Ordinary LeastSquares regression analysis of log-transformed data with R studio.^107^ We also tested whether these associations were biased by phylogenetic inheritance (inflation of Type I error) with Independent Contrast Analysis^101^ using the package Geiger^108^ of the R studio.^107^ To do this, we used a carnivoran supertree^102^ and we added *Miracinonyx* from previous sources^42^ and using Mesquite.^103^ Those species not present in our dataset were pruned using the R package Ape.^109^ In order to maximize sample size in bivariate regression analyses and not compromise statistical significance due to low sample sizes, we used the values of TEv and ACv for the species sampled by previous authors^55^ and we averaged the values of TEv and ACv for those species present in both datasets.

Our limited CT dataset precluded us to compute a bivariate regression analysis between paranasal sinuses volume against skull volume in order to explore quantitively whether *M. trumani* exhibits an enlarged paranasal sinus as in the case of *A. jubatus.* Therefore, we calculated a ratio between both variables and we qualitatively explored sinus volume in *M. trumani* relative to other felids present in the sample.

#### Quantifying brain shape

To quantify brain shape differences between *M. trumani* and other felid species, we digitized a set of 20 landmarks in 3D from 13 felid endocast (Figure 6). The landmarks were subject to Procrustes superimposition^110^ to remove the effects of size, rotation, and translation. The Procrustes coordinates were subject to Principal Components Analysis (PCA). To evaluate allometric effects, we regressed the Procrustes coordinates onto the logarithm of Centroid size using multivariate regression analysis.^96^ Afterwards, Procrustes coordinates and size-free residuals of the aforementioned regression were both analyzed with Principal Components Analyses using covariation. The geometric morphometric analyses were computed using MorphoJ.^111^

## Data Availability

• The 3d endocast generated in this study have been deposited to Figshare: https://figshare.com/s/b1366f7e7aef6317c62e. • Data of regional and total brain volumes and species body masses are provided in Table 1. • Any additional information required to reanalyze the data reported in this paper is available from the lead contact upon request. • This paper does not report original code.
