# Supplementary material for: The brain of the North American cheetah-like cat Miracinonyx trumani
Source: iScience. 2022 Nov 25;25(12):105671. doi: 10.1016/j.isci.2022.105671 (PMC9758517; doi:10.1016/j.isci.2022.105671)

iScience, Volume *25*

Supplemental information

The brain of the North American cheetah-like cat *Miracinonyx trumani*

Borja Figueirido, Alejandro Pérez-Ramos, Anthony Hotchner, David M. Lovelace, Francisco J. Pastor, and Paul Palmqvist

SUPPLEMENTARY INFORMATION

Table S1 (Related to Star Methods). Data acquisition of CT scans and resolution of each set of images through the bicubic technique of resampling. Pixel and Voxel sizes are in mm. Abbreviations: kV, kilovoltage;A, amperes; FOV, field of view.

Specimens	Number code registrer	kV	A	FOV	Pixel size	Voxel size original	Voxel size resampled
Acinonyx jubatus raineyii male	FMNH 29635	420	180	131	512X512	0.26 x 0.26 x 0.48	0.25 x 0.25x 0.25
Acinonyx jubatus raineyii female	FMNH 127834	420	180	131	507x402	0.25 x 0.25 x 0.46	0.25 x 0.25x 0.25
Acinonyx jubatus female	UWZS 23961	120	250	222	512X512	0.43 x 0.43 x 0.31	0.31 x 0.31 x 0.31
Acinonyx jubatus male	VU 6075	120	95	265	512X512	0.52 x 0.52 x 0.31	0.31 x 0.31 x 0.31
Acinonyx jubatus female	VU 6394	120	95	265	512X512	0.52 x 0.52 x 0.31	0.31 x 0.31 x 0.31
Puma concolor indet	UWZS 32281	120	300	179	512X512	0.35 x 0.35 x 0.31	0.31 x 0.31 x 0.31
Puma concolor male	VU 409	120	160	250	512X512	0.49 x 0.49 x 0.31	0.31 x 0.31 x 0.31
Puma concolor female	VU 3087	120	160	250	512X512	0.72 x 0.72 x 0.31	0.31 x 0.31 x 0.31
Puma concolor female	LACM 085440	420	180	142	504X384	0.28 x 0.28 x 0.50	0.28 x 0.28 x 0.28
Puma concolor male	LACM 087430	420	180	156	503X372	0.30 x 0.30 x 0.50	0.30 x 0.30 x 0.30
Panthera leo male	VU 6080	120	106	319	512X512	0.62 x 0.62 x 0.31	0.31 x 0.31 x 0.31
Panthera leo female	VU 2685	120	75	288	512X512	0.56 x 0.56 x 0.31	0.31 x 0.31 x 0.31
Lynx rufus male	OUVC 9576	80	450	250	1072x728	0.09 x 0.09 x 0.09	0.09x 0.09 x 0.09
Neofelis nebulosa male	USNM 282124	419	180	194	1024x1024	0.19x 0.19 x 0.25	0.18x 0.18 x 0.18
Miracinonyx trumani	KUVP 51277	120	250	143	512X512	0.28 x 0.28 x 0.31	0.28 x 0.28 x 0.28

Figure S1 (Related to Figure 3). Curvature map topology of endocasts. *A. jubatus* (UWZS 23961), *P concolor* (VU 409) and *M. trumani* (KUVP 51277) are shown as examples.

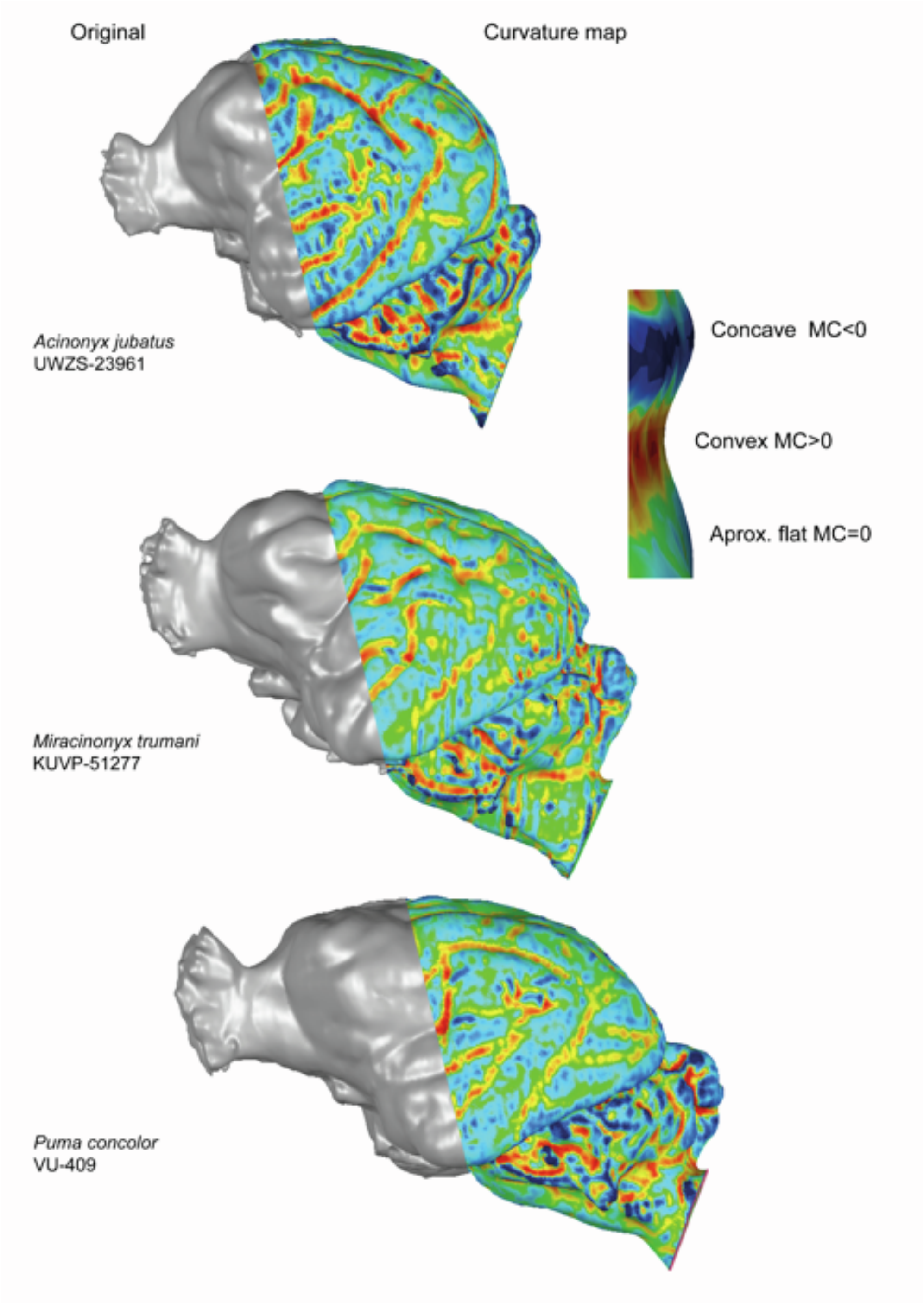

Figure S2 (Related to Figure 3). Topological deviation analysis in *A.jubatus* (UWZS-23961), *P concolor* (UWZS-32281) and *M. trumani* (KUVP-51277).

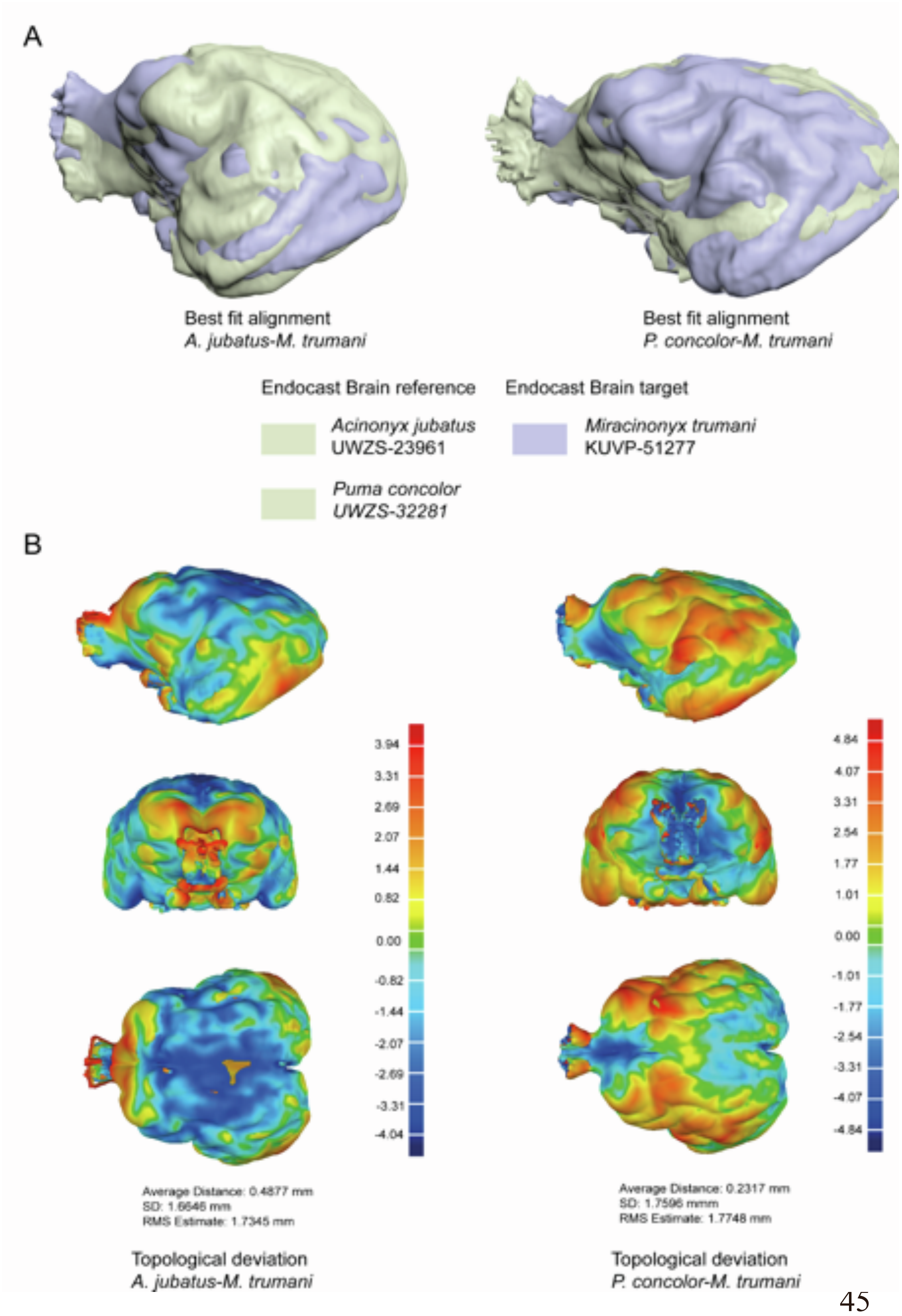

Supplement: Document S1. Figures S1 and S2 and Table S1 [file mmc1.pdf]
